# Fifty Years at the Brompton Hospital for Consumption

**Published:** 1905-09-23

**Authors:** 


### FIFTY YEARS AT THE BROMPTON HOSPITAL FOR CONSUMPTION.

FIFTY years ago, when Mr. Theobald, the secretary, who has ust retired, commenced his life-work at the Consumption Hospital, there was accommodation for only 180 patients in London and for a few more at a branch institution in Bournemouth, which is now a separate establishment. In 1882, owing to a bequest of £100,000 from Miss Read, the committee were able to increase the accommodation to 318 beds by the erection of the large building situated opposite the original hospital in the Brompton Road. The newer part also contains out-patients' department, mortuary, chapel, Röntgen ray room, and other special departments. In 1900 a very fine nurses' home was built, and in 1904 a sanatorium and convalescent home was opened near Frimley, this bringing up the capacity of the establishment to 418 beds.

These great structural developments only represent one side of the progress made at the Brompton Hospital during Mr. Theobald's connection with it. The modern medical treatment of consumption was adopted there about six years ago. Windows, hitherto hermetically sealed, were thrown open day and night, and hot water heating apparatus was superseded by coal fires in open fireplaces. Groups of patients are now to be seen under the shady trees in the hospital grounds, and out-door treatment is carried out as far as possible. There is a freshness and airiness about the whole building which it is surprising to find in the closely populated district of Brompton. It might have been expected that the public who have so enthusiastically supported the movement to treat consumption entirely in the open air and in the country should neglect a hospital situated in the metropolis. But those in charge of the Brompton Hospital promptly responded to the requirements of to-day, and by adding to its system a country sanatorium they preserved for it the right to be still regarded as the premier hospital for consumption. The Brompton Hospital has always been a favourite with the public and in Royal circles. It will be remembered that Queen Alexandra, when Princess of Wales, herself on one occasion assisted to entertain the patients by playing to them. The personal interest which is markedly shown in the hospital by benefactors and benefited alike is not a little due to the spirit of kindness and courtesy which prevails amongst the official staff. Mr. Theobald was eminently fitted for the post he occupied. His powers of organisation and discipline have for many years been apparent throughout the institution of which he was so justly proud. At the same time he never lost sight of the social requirements at such an institution. He realised the importance of encouraging and awakening interest in the hospital, and contributors, visitors, patients, and their friends received from him a courteous reception. Those who came again, even after some interval of time, were often surprised and pleased to find their identity known, for Mr. Theobald possesses that very useful gift of a memory for faces. The hospital is to be congratulated on the fact that Mr. Theobald has been succeeded by one trained under his guidance, and who will doubtless maintain the excellent tone which has been so conspicuous a feature there. Mr. Theobald has left the Brompton Hospital a very complete organisation, but he would feel a greater satisfaction in his well-earned leisure were he assured that the public would speedily testify to their appreciation of the increasing efforts the hospital has made to meet the demands upon it. The Frimley Sanatorium needs an income of £10,000 for its up-keep, and the requirements at Brompton are greater than ever they were. Glancing at a report of about thirty years ago and comparing it with one of the past year, we see nevertheless that very material economies have been effected. Thus the meat bill in 1876, when the patients numbered 1,222, was £2,270. In 1904 the patients counted 1,771, yet the meat bill only amounted to £1,819. Mineral waters in 1876 cost £112, in 1904 ice and mineral waters cost only £30. It is not to be supposed that the diet of the patients is any less generous than formerly, seeing that medical opinion has not altered in this respect. We must therefore conclude that very much more care is expended on the purchase of provisions. A study of the report of 1876 confirms us in this conclusion. Here we see that 13s. was paid for 1 cwt. of potatoes, 4s. for a fowl, and 1s. 8d. for a rabbit, 2s. 5d. per pound for tea, prices which would not be entertained in the present day, in connection with so large a household as at Brompton. But although we cannot look for a reduction in expenditure, seeing that no change of treatment in regard to diet has taken place, in respect to wine and spirits we see that in 1876 the expenditure under this head amounted to £760, whilst in 1904 it had fallen to £140. But in spite of the most careful management in the commissariat department, modern requirements have almost doubled the cost of administration at the Brompton Hospital, besides which the Convalescent Home at Framley has now to be provided for. Formerly the Brompton Hospital was one of the very few homes open to what were regarded as hopeless cases. In the appeal which accompanied the report of 1877 we read: "The plea on which the consumptive patient is refused admission into other institutions is the lingering nature and *almost certain fatality of his disease*." The hospital for consumption was in fact regarded either as a home for the dying, or a place of sojourn under more pleasant and favourable surroundings than that afforded by a poor home, to which, after a time of temporary improvement, some of the patients were returned. It may be therefore fairly assumed that the smaller death-rate at Brompton during recent years is not solely due to improved treatment—but also to the fact that cases almost *in extremis* are no longer received. The modern belief in recovery of the consumptive has caused patients in the early stages to be received almost to the exclusion of the hopeless cases. Mr. Theobald told us of the pain he had experienced through the necessity to refuse patients when beyond the hope of recovery. These generally come from the poorest homes, where there is neither money nor time to spend on the sufferer. In spite of all that medical science can do these hopeless cases of tuberculosis will exist in our midst, and need our care. The Brompton Hospital has done a great work of alleviation in the past. In its delightful wards kindness and care have soothed the lot of thousands of sufferers, for whom, in spite of the hopeless nature of their malady, physicians have made ceaseless efforts. Now in the light of modern science the institution has entered with faith and hope into the task of conquering the disease. "The almost certain fatality of the disease" acts no longer as the open sesame. The hospital is no longer a home for the dying. The public believe in the sanatorium rather than the hospital, and their support will therefore naturally be given to those institutions which adopt the principles of sanatorium treatment. Nevertheless, it is not impossible that a revolution of feeling will occur some day. It will be recognised that a percentage of fatal cases of tuberculosis must occur especially amongst the very poor, and public sympathy will return and provide care and comfort for these, such as was once so freely available at the Brompton Hospital. An institution admitting cases recognised as hopeless is most suitably situated in the city whence most of its patients come. For the incurable pure air is not so essential as comfort and the company of friends. With this consideration it is certain that there will always be a useful mission for such an asylum as the Brompton Hospital, should medical and public opinion demand that curative treatment should only be carried out in the country.

